# Increased flagellation enhances bacterial migration against and across flow

**DOI:** 10.64898/2026.10.16.759531

**Published:** 2026-10-16

**Authors:** Farhad Javi, Seiga Yanagisawa, Navish Wadhwa

## Abstract

Rheotaxis enables bacteria to swim against fluid flow, influencing their dispersal and colonization of surfaces. Although flagella generate the propulsion required for swimming, whether having more flagella improves rheotaxis remains unclear. Here, we varied flagellation using an *Escherichia coli* strain with tunable flagellar expression and tracked near-surface swimming under controlled microfluidic flow. Greater flagellation increased swimming speed without flow, reduced downstream transport under flow, and increased the fraction of tracks moving upstream. More flagellated cells also swam upstream for longer before switching direction. Greater flagellation also enhanced directed migration across the flow, suggesting that flagellar number alters the balance between propulsion, upstream alignment, and chiral turning near surfaces. A near-surface swimming model, in which flagellar number affects the swimming speed and the tilt of the cell toward the surface reproduced the trends in downstream transport, upstream swimming, and lateral migration. These results identify flagellar number as a determinant of how bacteria resist downstream transport and migrate across near-surface flows.

## Full Text Availability

The license terms selected by the author(s) for this preprint version do not permit archiving in PMC. The full text is available from the preprint server.

